# Emerging roles and potential application of PIWI-interacting RNA in urological tumors

**DOI:** 10.3389/fendo.2022.1054216

**Published:** 2023-01-17

**Authors:** Jingcheng Zhang, Wentao Zhang, Yuchao Liu, Man Pi, Yufeng Jiang, Ailiyaer Ainiwaer, Shiyu Mao, Haotian Chen, Yuefei Ran, Shuwen Sun, Wei Li, Xudong Yao, Zhengyan Chang, Yang Yan

**Affiliations:** ^1^ Department of Urology, Shanghai Tenth People’s Hospital, School of Medicine, Tongji University, Shanghai, China; ^2^ Urologic Cancer Institute, School of Medicine, Tongji University, Shanghai, China; ^3^ Department of Pathology, Shanghai Tenth People’s Hospital, School of Medicine, Tongji University, Shanghai, China; ^4^ Cancer Institute, Xuzhou Medical University, Xuzhou, China; ^5^ Center of Clinical Oncology, the Affiliated Hospital of Xuzhou Medical University, Xuzhou, China

**Keywords:** urological tumors, piRNAs, PIWI, non-coding RNA, application

## Abstract

The piRNA (PIWI-interacting RNA) is P-Element induced wimpy testis (PIWI)-interacting RNA which is a small molecule, non-coding RNA with a length of 24-32nt. It was originally found in germ cells and is considered a regulator of germ cell function. It can interact with PIWI protein, a member of the Argonaute family, and play a role in the regulation of gene transcription and epigenetic silencing of transposable factors in the nucleus. More and more studies have shown that piRNAs are abnormally expressed in a variety of cancer tissues and patient fluids, and may become diagnostic tools, therapeutic targets, staging markers, and prognostic evaluation tools for cancer. This article reviews the recent research on piRNA and summarizes the structural characteristics, production mechanism, applications, and its role in urological tumors, to provide a reference value for piRNA to regulate urological tumors.

## Introduction

With the in-depth study of RNA, it is recognized that only 2% to 3% of the human genome’s genes are transcribed and translated to proteins (1, 2). RNA transcribed from genes can be divided into two types where one is messenger RNA (mRNA) which transcribes from a strand of DNA as a template and thereby carries genetic information to guide protein synthesis while another is non-coding RNA (ncRNA) (3–5). Although, ncRNA cannot encode the synthesis of proteins still it has received extensive attention because of its role in cell function as well as disease etiology (6–8).

The ncRNA has been shown to play a key role in human carcinogenesis, as evidenced by several studies (9). Based on the molecular size of ncRNAs, they can be categorized into long-chain non-coding RNA (lncRNA) and short-chain (small non-coding RNA, sncRNA). The transcript of lncRNA is longer than 200 nucleotides (10). Contrarily, sncRNAs consist of microRNAs(miRNAs), short interfering RNAs(siRNAs), and PIWI-interacting RNAs (piRNAs) and so on (11). At present, the sncRNAs that have been deeply studied in tumors include miRNAs, siRNAs, and piRNAs. The lengths of these three RNAs are not equal where the length of miRNA is about 22nt, siRNA about 20-25nt, and piRNA is the longest at about 24-32nt. Unlike miRNAs and siRNAs that are derived from double-stranded RNA or RNA with folded structures, piRNAs are derived from single-stranded RNA precursors (12–14). Structurally, only siRNAs are double-stranded RNAs, piRNAs and miRNAs are single-stranded RNAs, and piRNAs are not known to have any conserved secondary structures or sequence motifs (15–17). The piRNAs can bind to PIWI proteins and participate in transposon control, germline development, spermatogenesis, and many other roles. The miRNAs regulate mRNA degradation or translation inhibition post-transcriptionally with partial mismatched sequences, while siRNAs have a natural defense against viruses as well as transposons and also aid in regulating post-transcriptional mRNA degradation with sequences of perfect match (18–20)(**Table 1**). Among the above three sncRNAs, the research on piRNA is getting deeper and deeper at this stage. According to the current database search, about 23439 piRNAs (21) have been identified from the human genome, which less than the total number of miRNAs currently studied (~ 35000) (22). This suggests that piRNAs may have several significant functions in gene regulation making them regarded as significant cell biology mediators.

**Table 1:** Comparison of the three sncRNAs.

Charateristics	piRNA	miRNA	siRNA
**Length**	About 24-32nt	About 22nt	About 20-25nt
**Source**	Single-stranded RNA precursor	Double-stranded RNA or RNA with folded structure	Double-stranded RNA or RNA with folded structure
**Space structure**	Single stranded RNA	Single stranded RNA	Double stranded RNA
**Secondary structure**	NA	Yes	Yes
**Effects**	It binds to PIWI protein and participates in transposon control, germline development, spermatogenesis, etc.	Post-transcriptional regulation of mRNA degradation or translational repression with partially mismatched sequences	Natural defense against viruses and transposons, regulating mRNA degradation post-transcriptionally with perfectly matched sequences

NA means Not Available.

Although various studies have been conducted on piRNA the research on piRNA association with different diseases is still in its earliest stages and more thorough work is needed to be done for studying the biological functions as well as roles of piRNA in the field of human health, disease, and its main role to regulate epigenetics, for controlling various biological processes. The most studied role is its relationship with tumor theories regulating tumor growth, and invasive and distant metastasis. At present, a series of differentially expressed piRNAs have been found in gastric cancer, liver cancer, breast cancer, and lung cancer along with other tumors such as piR-651, piR-Hep1, and piR-021285 (23–27). These piRNAs not only regulate the proliferation, invasion, and migration of cancer cells by affecting either upstream or downstream signaling pathways, protein expression, epigenetic modification, and other pathways but also are expected to be used as markers for non-invasive diagnosis, drug treatment targets and prognostic biomarkers in clinical aspects.

The function of piRNA in tumors is constantly proposed, researchers have also found that piRNA is closely related to urological tumors. Now, some studies on the relationship between piRNA and urological malignancies have been carried out (28–31). The tumors of the urinary system mainly include renal carcinoma, bladder cancer, prostate cancer and testicular cancer. The incidence rate of prostate cancer among elderly men is higher where the PSA level needs to be closely detected (32, 33). Presently, castration-resistant prostate cancer still possess difficulty in treatment. There is also a lack of specific tumor markers for kidney and bladder cancers. At present, the non-invasive diagnosis and patient prognosis evaluation of urological tumors are still a hot topic in basic research and clinical transformation where piRNA is expected to become a sensitive and reliable marker for diagnosis and prognosis of urological tumors. This paper reviews the research performed on piRNA in the past 10 years while summarizing the structural characteristics, biosynthetic process, its role in urological tumors, and possible clinical significance.

## PiRNA

### PiRNA and PIWI proteins

The piRNA is a class of 24-32nt ncRNAs isolated from mammalian germ cells. In mammalian systems, they were formally designated as piRNA in 2006 due to their association with the PIWI subfamily of Argonaute proteins (34–38). The piRNA accumulates at the beginning of meiosis or during spermatogenesis thus ensuring their involvement in reproduction as well as in regulating fertility (36, 37, 39, 40). piRNA forms a piRNA/PIWI complex by binding with PIWI proteins, and then plays a series of roles. It also plays a gene silencing role *via* PIWI-dependent transposon silencing, epigenetic control, gene and protein regulation, genome rearrangement as well as maintaining germ stem cells (41–43).

The PIWI proteins have been identified first in Drosophila (44) and are also reported to be involved in germline stem cell maintenance as well as self-renewal (45). PIWI proteins belong to the PIWI subfamily, which further belongs to the Argonaute protein family. Five Argonaute proteins identified in Drosophila are Ago1, Ago2, Ago3, PIWI, and Aubergine (Aub). In addition, the PIWI subfamily shows highly conserved structural and functional properties in Drosophila, mouse (MILI, MIWI, and MIWI2), human (HILI (PIWIL2), HIWI (PIWIL1), HIWI2 (PIWIL4), and HIWI3 (PIWIL3)). There are three Argonaute proteins in germ cells, namely Ago3, PIWI, and Aub (46, 47). As a nuclear protein, PIWI is crucial for retrotransposon silencing as well as regulation of male germ line migration (48). In addition, PIWI is also involved in the formation of germ cells (45, 49). Knockout or mutation of PIWI protein may lead to sperm development defects (48). Many studies have established the PIWI protein’s function in cancer, such as PIWIL2 can affect tumor development by inhibiting apoptosis and promoting cell proliferation (50). Many studies have found PIWI proteins can affect the occurrence and development of a variety of cancers (50–52).

### Structural characteristics of piRNA

The piRNA is a kind of small RNA with a single-stranded length of 24-32nt, most of which are 29-30nt (53, 54). About 76% of miRNAs have uracil at the 5′ ends, while piRNAs also have a strong tendency to uracil at the 5′ ends (about 86%) (55). The distribution of piRNAs on chromosomes is extremely uneven. In mice, they are mainly distributed on chromosomes 17, 5, 4, and 2 but rarely on chromosomes 1, 3, 16, 19, and X also basically not on chromosome Y (36). In addition, piRNA mainly exists in the gene spacer region, but rarely in the gene region or repeat sequence region. They are mainly clustered at relatively short genomic loci of 1-100kb and contain 10-4500 small RNAs (55). Because piRNAs are distributed in clusters, and each of them has almost the same orientation, it indicates that the same cluster of piRNAs may originate from the same long initial transcript, but some clustered piRNAs will suddenly change orientation, which indicates that these bidirectional clustered piRNAs may be transcribed from the same promoter in different ways (55).

### Biosynthesis of piRNA

Since the discovery of piRNA, the source of such small RNAs has been one of the research focuses of researchers. A current study shows that the synthesis of mature piRNA needs to go through two steps, namely transcriptional production of piRNA precursors and post-transcriptional production of mature piRNA.

#### Production of piRNA precursors

The vast majority of precursors of piRNA generate from the piRNA cluster’s genetic regions that are divided into single-stranded clusters and double-stranded clusters. The piRNA clusters in Drosophila ovarian somatic cells is a single-stranded clusters which generate precursors localized to only one strand, while piRNA clusters in germ cells is the double-stranded clusters which generate precursors localized to two genomic strands. The precursors of piRNA are generated by single-stranded clusters produced by conventional unidirectional transcription in all animals that produce piRNA being detected so far. By definition, double-stranded cluster piRNAs create both sense and antisense piRNA precursors independent of transposon orientation (56, 57). At present, double-stranded clusters transformed from two DNA strands have only been found in some insects (58–60), but they are likely to exist in other organisms. Protein coding genes can generate some piRNA precursors from their 3’UTRs (61).

Transcription of Drosophila single-stranded piRNA clusters is achieved through the formation of RNA polymerase II pre-initiation complexes in inhibitory heterochromatin. The single-stranded cluster transcription is identical to classical mRNA transcription. The single-stranded cluster contains a transcription-related H3K4me2 marker at the promoter. In addition, the piRNA precursors of single-stranded clusters are those with a “cap” at the 5’ end and a “tail” at the 3’ end (60, 62).

**Figure 1:**
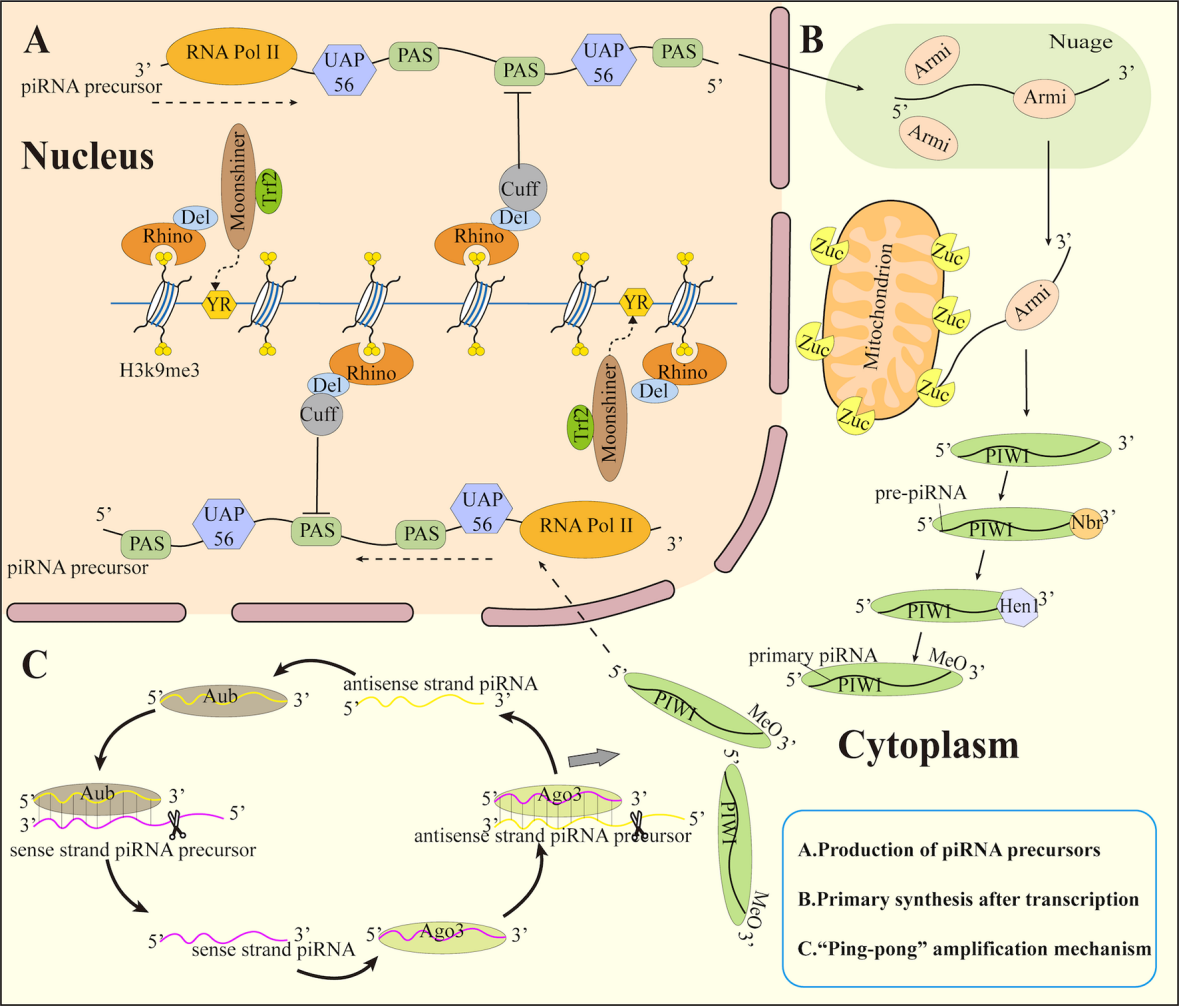
Biosynthesis of piRNA. **(A)** Production of piRNA precursors **(B)** Primary synthesis after transcription **(C)** ”Ping-pong” amplification mechanism. Trf2, TATA box-binding protein-related factor 2; Del, Deadlock; Cuff, Cutoff; YR, pyrimidin purine; PAS, polyadenylation signal sequence; Armi, Armitage; Zuc, Zucchini; Hen 1, methyltransferase Hen1; Nbr, Nibbler; MeO, methoxyl.

Several proteins like RNA Pol II, Rhino-Deadlock-Cutoff complex(RDC complex), Moonshiner, TATA-box binding protein-related factor 2 (Trf2), and UAP56 participate in the transcription of double-stranded clusters into sense and antisense piRNA precursors. Among these, the H3K9me3 inhibitory marker on the double-stranded cluster was recognized by Rhino (63). It also forms a complex with Deadlock (Del) as well as Cutoff (Cuff) [48]. To initiate promoter-independent transcription, Rhino-Del recruits as well as Trf2 to the pyrimidin purine (YR) element (64), in a process that does not require RNA polymerase to bind the promoter. The RDC complex ensures the elongation of the transcriptional precursor piRNA by inhibiting the splicing and termination of the polyadenylation signal sequences (PAS) within the cluster. After that, the piRNA precursor is transferred to the Nuage outside the nucleus through UAP56. Although the precursor obtained in this way has no polyadenylate tail, Cuff can also prevent the exonuclease from cutting the 3’ tail of the precursor (**Figure 1**).

#### Production of mature piRNA

Following transcription, mature piRNAs are produced by two main mechanisms: the primary synthesis of piRNAs and the “ping-pong” amplification mechanism (**Figure 1**).

##### Primary synthesis after transcription

The piRNA precursor delivered to the outside of the nucleus enters a membrane-free electron-dense structure in the cytoplasm (65), which contains most of the proteins that process piRNA precursors. It is named Nuage (66) in Drosophila germ cells and Yb body (67) in Drosophila ovary somatic cells. It also exists in the outer mitochondrial membrane of piRNA-producing cells at all stages. This membrane-free electron-dense structure may aid in the localization of certain proteins or the protection of piRNA precursors from nucleases. Its function might potentially block mRNA as well as lncRNA to enter the piRNA synthesis pathway.

In Drosophila ovarian germ cells, piRNA precursors are transported by Armitage (Armi) in Nuage to the mitochondrial surface and cleaved to pre-piRNA containing 5’ monophosphate by mitochondrial associated endonuclease Zucchini (Zuc) (68). The pre-piRNA was then loaded onto the PIWI protein and trimmed at the 3’ end with the exonuclease Nibbler (Nbr). Meanwhile, the short RNA 2’-O-methyltransferase Hen1 methylates the newly produced 2’ hydroxyl group at the 3’ end (69). This process is known as primary piRNA biogenesis, and the piRNA produced in this manner are known as primary piRNA.

##### “Ping-pong” amplification mechanism

The amplification of piRNA is initiated by primary piRNA and progresses *via* the involvement of Ago3 and Aub proteins. A piRNA/Aub complex is formed by bind of Aub to the antisense strand piRNA precursor and also cleaves the use strand piRNA precursor to generate a sense piRNA bound to Ago3. Subsequently, Ago3 binds the sense strand piRNA precursor to form piRNA/Aog3 and cleaves the antisense strand piRNA precursor to generate antisense piRNA loaded onto Aub (42, 70, 71). This round of repeat cutting creates a huge number of piRNAs in a short period. This mode is called the secondary biogenesis of piRNA or the “ping-pong” amplification mechanism. PIWI proteins then bind to these piRNAs and transport them back to the nucleus, where they silence target genes.

### Biological functions of piRNA

The piRNA sequences and functions vary greatly among different species. According to current research, its possible biological functions include mediating transposon silencing, epigenetic control (72), gene as well as protein regulation (73), and participating in spermatogenesis (74). However, at present, only the mechanism that piRNA can mediate transposable elements (TEs) silencing has been studied in depth and has become a consensus (75–78).

At the transcriptional level, piRNA regulates TEs silencing through DNA methylation modification. In the nucleus, piRNA and PIWI regulate DNA methyltransferase (DNMT) directly modifying chromatin structure and histones where transcriptional initiation is inhibited by the methylation of CpG islands by DNMT (79). DNMT guided by PIWI proteins binds to transposons or target genes, thus completing transposon or target gene silencing. However, the precise mechanism *via* which piRNA and PIWI proteins regulate the expression of DNMT protein in this process is poorly understood. piRNA and PIWI proteins also interact with histone methylation processes and influence histone lysine methylation (H3K and H4K). They recruit histone methyltransferases to target transcripts to inhibit transcription (80, 81). But so far, the relationship between histone modification and piRNA has not been solved.

The piRNA can also silence TEs through chromosomal heterochromatinization. piRNA can recruit PIWI, HP1a, and Su(var) 3-9, but also make H3K9me2/3 enrichment and RNA polymerase II association reduced (82). It was found that PIWI specifically interacts with heterochromatin protein 1a (HP1a), which is the main participant in heterochromatin gene silencing. Its main binding interaction involves HP1a binding to the PxVxL type motif of the PIWI N-terminal domain. The PxVxL type motif is necessary for Drosophila for normal silencing of transgenes embedded in heterochromatin (83). This study shows that the combination of piRNA, PIWI protein, and heterochromatin protein plays a direct role in determining the epigenetic state of the Drosophila genome.

piRNA and PIWI proteins stimulate mRNA degradation at the post-transcriptional phase *via* mRNA degradation pathways. The main way to attenuate mRNA and hinder translation involves shortening of mRNA polyadenylation tail, in a process named deadenylation (84).

In addition to transposon silencing, piRNA has also become a hot spot in gene regulation recently. For example, Gao et al. (73) found cardiac hypertrophy associated with piRNA (CHAPIR), which can promote cardiac pathological hypertrophy and remodeling through the regulation of genes and proteins. A loss of CHAPIR considerably reduced cardiac hypertrophy and restored cardiac function, while mice with pressure overload of CHAPIR mimetics had a response to pathological cardiac hypertrophy. Mechanistically, the CHAPIR-PIWIL4 complex directly interacts with METTL3 to block m6A methylation of PARP10 mRNA transcripts, thereby upregulating the expression of PARP10. CHAPIR-dependent increase of PARP10 in turn promotes single ADP-ribosylation of GSK3β and inhibits its kinase activity, which leads to accumulation of nuclear NFATC4 and progression of cardiac pathological hypertrophy. This study shows that changes in the expression level of piRNA may affect the methylation of gene transcripts and then the expression level of genes, which also provides new insights into piRNA as a human gene regulator.

piRNA also plays a crucial role in spermatogenesis. Dai et al. (74) found a similar mechanism to be responsible for the activation of the translational part of spermatogenic mRNA to coordinate the morphological transformation of sperm. This kind of action requires specific base pairing interaction between piRNA and target mRNA in the 3’UTR, which stimulates translation by interacting with AU-rich cis-acting regions and thereby nucleates the MIWI/piRNA/eIF3f/HuR super-complex in a developmental stage-specific manner. This finding reveals the crucial role of the piRNA system in activating translation activation and proves that sperm cell development is functionally necessary.

**Figure 2:**
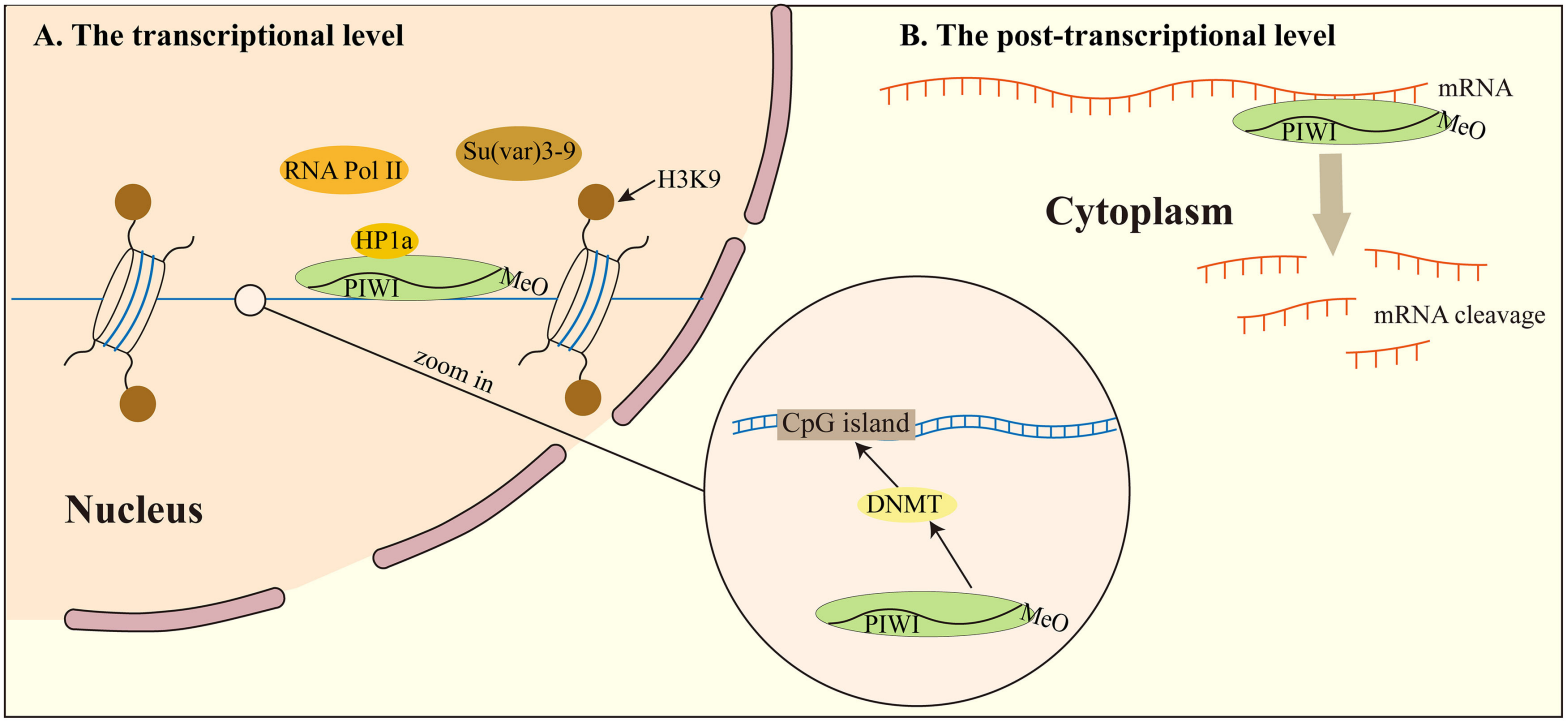
piRNA regulates transposon silencing. **(A)** at the transcriptional level, piRNA regulates transposon silencing through DNA methylation modification. What’s more, the piRNA can also silence TEs through chromosomal heterochromatinization. **(B)** at the post-transcriptional level, piRNA recognizes a target mRNA and mediates its degradation by deadenylating or cleaving the mRNA. DNMT, DNA methyltransferase.

Obviously, piRNAs play various roles in several cellular processes, therefore it is currently difficult to investigate their potential functions. However, we believe that soon, other piRNA functions will be revealed to deepen our understanding of piRNA (**Figure 2**).

## Study of piRNA in urological tumors

Urological tumor is a common disease worldwide, which mainly includes renal carcinoma, bladder cancer, prostate cancer and testicular cancer. Although they are all diseases with high incidence rate and mortality, non-invasive diagnosis and evaluation of patient prognosis are still a major problem in medicine at this stage. Existing diagnostic techniques such as liquid biopsy, including circulating tumor cells (CTCs), circulating tumor DNA (ctDNA) and extracellular vesicles (EVs), have been demonstrated their potential diagnostic value in urological tumors (85–88). And in prostate cancer, the introduction of genomic biomarkers has dramatically improved detection, prognosis, and risk assessment (89). But now we can detect the differentially expressed piRNAs in urological cancer to diagnose and evaluate the prognosis of patients, and piRNAs is expected to become a potential therapeutic target (**Table 2**).

**Table 2:** Expression and application of piRNAs in urological tumors.

piRNA types	Functions \ possible clinical applications	expression	References
Renal carcinoma			
piR-32051	Prognostic marker	Up regulation	(28)
piR-39894	Prognostic marker	Up regulation	
piR-43607	Prognostic marker	Up regulation	
piR-30924	Prognostic marker	Up regulation	(90, 91)
piR-38756	Prognostic marker	Up regulation	
piR-57125	Inhibit cancer cell proliferation, invasion, and migration, and can be used as a diagnostic tool and a therapeutic target	Down regulation	
piR-34536	Prognostic marker	Down regulation	(92)
piR-51810	Prognostic marker	Down regulation	
piR-823	Diagnostic tool	Down regulation	(93)
piR-823(urine)	Diagnostic tool	Up regulation	
piR-31115	Promote cancer cell proliferation, invasion, and migration, and can be used as a diagnostic tool and a therapeutic target	Up regulation	(94)
Bladder cancer			
piRABC(piRNADQ594040、piR-60152)	Inhibit cancer cell proliferation, invasion, and migration, and promote apoptosis, and can be used as a diagnostic tool and a therapeutic target	Down regulation	(95)
piR-5936(plasma exosomes)	Diagnostic tool	Up regulation	(29)
Prostate cancer			
piR-651	Related to hormone therapy for cancer	Up regulation	(96)
piR-823	Related to hormone therapy for cancer	Up regulation	
piR-001773	Promote cancer cell proliferation, invasion, and migration, and can be used as a diagnostic tool and a therapeutic target	Up regulation	(30)
piR-017184	Promote cancer cell proliferation, invasion, and migration, and can be used as a diagnostic tool and a therapeutic target	Up regulation	
piR-31470	Promote cancer cell proliferation, invasion, and migration, and can be used as a diagnostic tool and a therapeutic target	Up regulation	(97)
Testicular cancer			
PIWI/piRNA	The loss of this pathway is beneficial to the proliferation, invasion, and migration of cancer cells	Defect	(31, 98, 99)

PubMed and Web of Science were used for searching the articles related to urological tumors and piRNA in the past 10 years.

### Renal carcinoma

More than 400,000 people worldwide suffer from renal cell carcinoma (RCC) every year. The age of diagnosis is about 60 years old, and the number of men diagnosed is twice that of women (100). Renal cell carcinoma comprises various subtypes each with its distinct traits and the most frequent subtype (about 70% of cases) is clear cell renal cell carcinoma (ccRCC) (101). At present, it is difficult to make an early diagnosis of renal carcinoma, however, some investigations have revealed that piRNAs can be utilized as a possible marker of renal carcinoma as strongly associated with renal carcinoma metastasis and prognosis. Simultaneously, during different studies, researchers found some differentially expressed piRNAs in different samples (such as tissue, urine, serum, etc.) which may also provide a research basis for the non-invasive diagnosis of renal carcinoma.

Li et al. (28) found that piR-32051, piR-39894, and piR-43607 among the same cluster of piRNAs were overexpressed in ccRCC by RNA-seq analysis, and further confirmed that the overexpression of the three piRNAs was significantly related to ccRCC metastasis by using a paraffin tissue cohort of 68 samples. In addition, the researchers also demonstrated that their expression was significantly related to the advanced stage of ccRCC and the cancer-specific survival rate of patients. Busch et al. (90) found the relationship between three piRNAs and ccRCC metastasis and progression through piRNA microarray analysis technology. They used RT-qPCR to measure the piRNAs among non-metastatic (n = 76) as well as metastatic (n = 30) ccRCC tissues during nephrectomy, and then compared them with the corresponding piRNAs from normal kidney tissues (n = 77) and distant ccRCC metastatic tissues (n = 13). These findings revealed that piR-57125 expression in metastatic tumors was lower than that in non-metastatic tumors, while increased expression of piR-30924, as well as piR-38756, was found among metastatic tumors. The overexpression of piR-30924 as well as piR-38756 while a low expression of piR-57125 among metastatic primary tumors was found considerably correlated to tumor recurrence and overall survival.

The deregulation of piRNA can not only serve as a diagnostic marker of renal carcinoma metastasis but also directly regulate the metastasis and invasion of ccRCC through some signaling pathways. Du et al. (94) revealed piR-31115 to be upregulated in ccRCC tissues through epithelial-mesenchymal transition (EMT) and PI3K/AKT signaling pathways while promoting cell proliferation as well as invasion. After down-regulating the level of piR-31115 in this experiment, the epithelial marker E-cadherin protein expression was up-regulated along while the expression of interstitial markers vimentin and snail was decreased along with a significant decrease in the expression of AKT and PI3K phosphorylation. EMT is a process in which epithelial cells acquire mesenchymal characteristics. In cancer, EMT is related to tumor initiation, invasion, metastasis, and resistance to treatment while PI3K is a crucial oncogenic factor that regulates multiple pathways, including cell proliferation and invasion, as well as the EMT process. In addition, Ding et al. (91) reported down-regulation of piR-57125 in ccRCC tissues. After knocking down piR-57125, the experimenter found that the migration and invasion of ccRCC became faster, and overexpression of piR-57125 inhibited the metastasis of ccRCC. Simultaneously, the lung metastasis model *in vivo* validated similar results. Moreover, functionally CCL3 acts as a downstream target of piR-57125 where piR-57125 binds to CCL3 directly and inhibits ccRCC metastasis by down-regulating the AKT/ERK axis.

The expression of piRNAs may be different in different specimens, and its expression level is significantly related to the survival period of patients. Zhao et al. (92) extracted total RNA from renal cell carcinoma tissue, normal kidney tissue, serum of patients with renal cell carcinoma, and serum of patients with non-malignant diseases, and measured the expression of piRNAs isolated from cell mitochondria using quantitative real-time PCR. The results showed that the expression of piR-34536 and piR-51810 were down-regulated in renal cell carcinoma compared with non-malignant renal tissues, and the decrease of tissue piRNAs level was a significant independent predictor of the shortening of progression-free survival, cancer-specific survival, and overall survival of ccRCC patients. There was no difference in serum piRNAs levels between ccRCC patients and control subjects.

Liquid biopsy and non-invasive diagnosis of renal cell carcinoma have attracted more and more attention. There are also differentially expressed piRNAs in urine of patients with renal cell carcinoma, so urine may also become one of the non-invasive diagnostic points for renal cell carcinoma. However, the expression of a certain piRNA in the urine of ccRCC patients is very different from that in tumor tissues. Iliev et al. (93) revealed dysregulated expression of piR-823 among tumor tissues and urine of ccRCC, but the dysregulation results of the two were just the opposite. The expression of piR-823 has been down-regulated in tumor tissues and was positively correlated with poor prognosis. The researchers also measured the level of piR-823 in urine samples of 20 RCC patients and 15 healthy controls. The results showed that the level of piR-823 in urine samples of RCC patients was considerably higher in comparison to healthy controls (P = 0.0157). Although they are both piR-823, it is still unclear why their expression results are opposite in tissues and urine.

**Figure 3:**
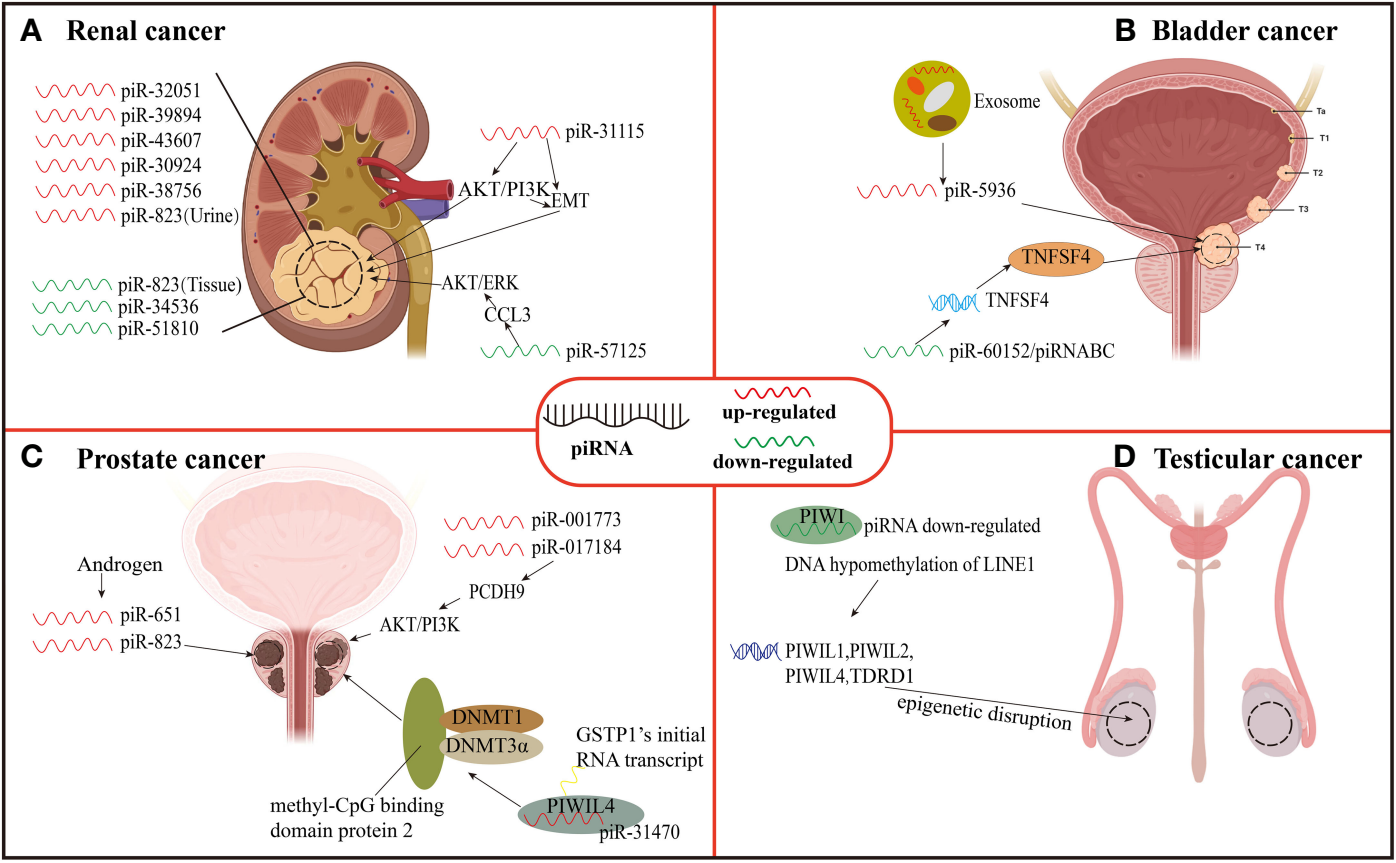
Expression and function of piRNAs in urinary neoplasms. **(A)** Renal carcinoma. **(B)** Bladder cancer. **(C)** Prostate cancer. **(D)** Testicular cancer.

At present, piRNAs are not completely optimized for clinical application, but they are possible to use differentially expressed piRNAs for early diagnosis and prognosis evaluation of patients with ccRCC in the future. In the above studies, among ccRCC tumor tissues the piR-32051, piR-39894, piR-43607, piR-30924, and piR-38756 were all up-regulated where this up-regulation was closely related to cancer metastasis and low specific survival rate which can be used as prognostic markers, while piR-31115 and piR-57125 can regulate ccRCC progression making them potential therapeutic targets for ccRCC. A down-regulation of piR-823 in tumor tissues of patients has been related to poor prognosis in tissues. Simultaneously, the down-regulated expression of piR-34536 and piR-51810 isolated from cell mitochondria can be used as one of the factors predicting the prognosis of patients. Urine piR-823 can be used as a target for non-invasive diagnosis of ccRCC (**Figure 3**).

### Bladder cancer

Bladder cancer is an aggressive tumor with high mortality. It is the most common malignant tumor in women and the fourth most common malignant tumor in men (102). There were an estimated 500, 000 new cases of bladder cancer diagnosed in 2019 and 200, 000 deaths from the disease (103). Some studies have found that piRNAs may affect the progress of bladder cancer. For example, Chu et al. (95) investigated global piRNAs expression in three cases of bladder cancer tissues and their neighboring normal tissues using piRNA microarray. The results showed that in bladder cancer tissues, 106 piRNAs were found to be up-regulated whereas 91 were down-regulated with piRNADQ594040 (piRABC and piR-60152) related to bladder cancer had the highest down-regulation fold change. In this study, the researchers found that the mRNA, as well as protein expression of TNFSF4 in normal bladder tissues adjacent to cancer, was higher in comparison to that among bladder cancer tissues. TNFSF4 is a binding partner of OX40, and in cancer treatment, the OX40-TNFSF4 pathway has been found to influence immune tolerance (104). Moreover, the TNFSF4 expression in cancer-free serum was found higher than that in bladder cancer serum (95). Therefore, the down-regulation of piRABC expression may promote cell proliferation, and colony formation and inhibit apoptosis of bladder cancer by affecting the TNFSF4 gene and thus down-regulating TNFSF4 protein. Thus, it can be seen that the TNFSF4 gene is a potential target gene of piRABC, and piRABC may also be a potential therapeutic target for bladder cancer.

Most of the diagnosis of bladder cancer relies on cystoscopy and pathological biopsy, which are time-consuming and labor-consuming, and patients have to bear great pain during the examination. Now, liquid biopsy of bladder cancer can alleviate the pain of patients and obtain a variety of analytes and predict the prognosis of patients, such as CTCs, ctDNA, circulating acellular tumor RNA (ctRNA), proteins, peptides, and metabolites (105). However, the piRNAs of plasma exosomes has not been measured yet. Exosomes (106) are small vesicles secreted or shed from the membrane by cells, which contain some cytoplasmic components, such as protein, mRNA, sncRNA, DNA, and piRNA contained therein will have profound significance in clinical diagnosis and treatment in the future. Sabo et al. (29) evaluated the expression level of sncRNA in plasma exosomes from 47 bladder cancer males and 46 healthy controls by next-generation sequencing. SncRNA profiles were compared with urine profiles from the same subject. Compared with the control group, piR-5936 was highly expressed in high-risk bladder cancer. It indicates that piR-5936 from plasma exosomes may be a diagnostic biomarker of bladder cancer, especially in the advanced stage of bladder cancer.

Although there are many studies on bladder cancer, so far, only a few researchers have studied the mechanism of piRNA and proliferation, invasion, and metastasis of bladder cancer. However, miRNA and siRNA, which belong to sncRNA like piRNA, have made rapid progress in the research on the mechanism of bladder cancer. In terms of clinical diagnosis, if the above three sncRNAs are combined, it may be more accurate for the detection and diagnosis of bladder cancer, but we still need to explore piRNAs and other sncRNAs in combination (**Figure 3**).

### Prostate cancer

Prostate cancer is a complex disease about 1,600,000 men are diagnosed with prostate cancer every year, and 366,000 die from it. This disease is heterogeneous and accounts for one of the major causes of cancer-related deaths worldwide. If prostate cancer can be detected early and actively treated, some patients with low and medium recurrence risk usually have a 10-year overall survival rate of 99% (107). There are various risk factors for the occurrence of prostate cancer, among which the level disorder of dihydrotestosterone and other androgen is inseparable from the incidence of prostate cancer (108). However, studies have found that the level of hormones can affect the level of piRNAs and thus affect the progression of prostate cancer. Oner et al. (96) treated androgen-dependent and androgen-independent prostate cancer cells (LNCaP and PC-3) with androgen, and after hormone treatment, piR-651 and piR-823 expression were increased in prostate cancer cell lines. The experimental results show that the increased expression of piR-651 and piR-823 may be related to the changes in hormone levels in the cancer microenvironment, and this result may also open a new idea for hormone treatment of prostate cancer.

In addition, some piRNAs present in prostate cancer cells can promote the proliferation, invasion, and metastasis of prostate cancer cells through specific pathways. Zhang et al. (30) revealed piR-001773 and piR-017184 overexpression among prostate cancer tissues. Protocadherin 9 (PCDH9) is down-regulated in prostate cancer cells and acts as a tumor suppressor. PCDH9 can binds to p85α which is a PI3K regulatory subunit. The PCDH9 down-regulation in prostate cancer cells leads to an enhanced AKT phosphorylation and activity thereof. The piR-001773 and piR-017184 regulate PCDH9 post-transcriptionally which promotes proliferation, invasion, and metastasis of prostate cancer cells and effectively promotes the growth of tumors *in vivo* and *in vitro*. In contrast, the down-regulation of piR-001773, as well as piR-017184, significantly inhibits tumor growth.

Glutathione S-transferase Pi1 (GSTP1) occurs in the early stage of prostate cancer through methylation inactivation and can be detected in up to 90% of prostate cancer (109). Functional inactivation of GSTP1 increases susceptibility to oxidative stress and increases the risk of progression of prostate cancer. On this basis, Zhang et al. (97) also discovered that piR-31470 may bind to PIWIL4 forming a PIWIL4/piR-31470 complex. This complex can bind GSTP1’s initial RNA transcript and recruit DNA methyltransferase 1, 3α, and methyl-CpG binding domain protein 2 to begin and sustain hypermethylation and inactivation of GSTP1. The data showed a piR-31470 overexpression mediated inhibition of GSTP1 levels and also increased susceptibility of human prostate epithelial RWPE1 cells to oxidative stress and DNA damage. This finding may present a novel treatment technique for prostate cancer epigenetic therapy by targeting piRNA. The above-mentioned piR-001773, piR-017184, and piR-31470 are all overexpressed in tumor tissues, and they can also promote the proliferation, invasion as well as migration of cancer cells through specific pathways. Due to their potential applications, CTCs, EVs, ctDNA and ctRNA of blood have been extensively studied as a part of liquid biopsies in prostate cancer (110). In the future, piRNAs are anticipated to be used as diagnostic tools and treatment targets for prostate cancer (**Figure 3**).

### Testicular cancer

The most common malignancy in young men between 14 to 44 years of age is testicular germ cell tumors (TGCT) in Western countries. There will be 9560 new cases and 410 deaths of TGCT in the United States in 2019. TGCT prevalence and incidence have been on the rise in the past two decades (111). piRNA was first found in germ cells and is also a key factor for germ cell maintenance. PIWI/piRNA pathway is not only critically involved in the development of the male germ line but also plays an important role in the occurrence and progression of testicular cancer.

In the study on the relationship between epigenetic disruption of PIWI/piRNA pathway and testicular cancer, Ferreire et al. (31) have found epigenetic abnormalities of PIWI protein and piRNA in testicular cancer. They found that in addition to testicular germ cell tumors, there was a decrease in piRNA expression and DNA hypomethylation of LINE1 (PIWI/piRNA target sequence) in primary seminoma and non-seminoma testicular tumors, and their reduction also led to epigenetic inactivation of PIWI-like protein (PIWIL1, PIWIL2, and PIWIL4) and their related TDRD1 protein gene in human testicular tumorigenesis. Rouge et al. (98) performed small RNA sequencing on 22 human TGCT samples from 5 histological subtypes, 3 carcinomas in situ, and 12 normal testis samples, and found that the expression of piRNA populations of all TGCT subtypes was globally absent compared with normal testis. These research data all show that the epigenetic disruption of the entire PIWI/piRNA pathway is indeed a sign of testicular tumor development.

In addition, the loss of the PIWI/piRNA pathway may also provide favorable conditions for the progress of TGCT. Gainetdinov et al. (99) used small RNA deep sequencing, qRT-PCR, and mining public RNA seq/small RNA-seq data to detect the PIWI/piRNA gene expression and piRNA biosynthesis of cells in the development stage of TGCT. Three types of samples were studied: 1. Healthy testis, 2. Testicular tumor paracancerous tissue and germ cell neoplasia *in situ* (GCNIS), 3. Matched TGCT samples. The result was that piRNA biogenesis was complete in germ cells of the healthy adult testis and adjacent tissues of testicular tumors. In contrast, there is a lack of PIWI/piRNA pathway gene expression and germline-like piRNA biogenesis in GCNIS and TGCT cells. However, there is no evidence that the PIWI/piRNA pathway is dysregulated in germ cells adjacent to TGCT, which contradicts its role as an oncogenic driver of TGCT development. However, *in vitro* experiments demonstrated that the PIWIL2/HILI short isoform may play a role in inhibiting transposon silencing, which can provide growth advantages for TGCT by ensuring its genomic integrity. Therefore, detecting the deletion of the PIWI/piRNA pathway can be used as an auxiliary means for the clinical diagnosis of testicular cancer (**Figure 3**).

### Rare urological tumors

Up to now, few researchers have studied the relationship between piRNAs and rare urological tumors, such as penis cancer. However, given that piRNAs have many potential applications in other tumors, we have reason to believe that piRNAs still have the potential as a diagnostic tool and therapeutic target in rare urological tumors.

## Conclusion

**Figure 4:**
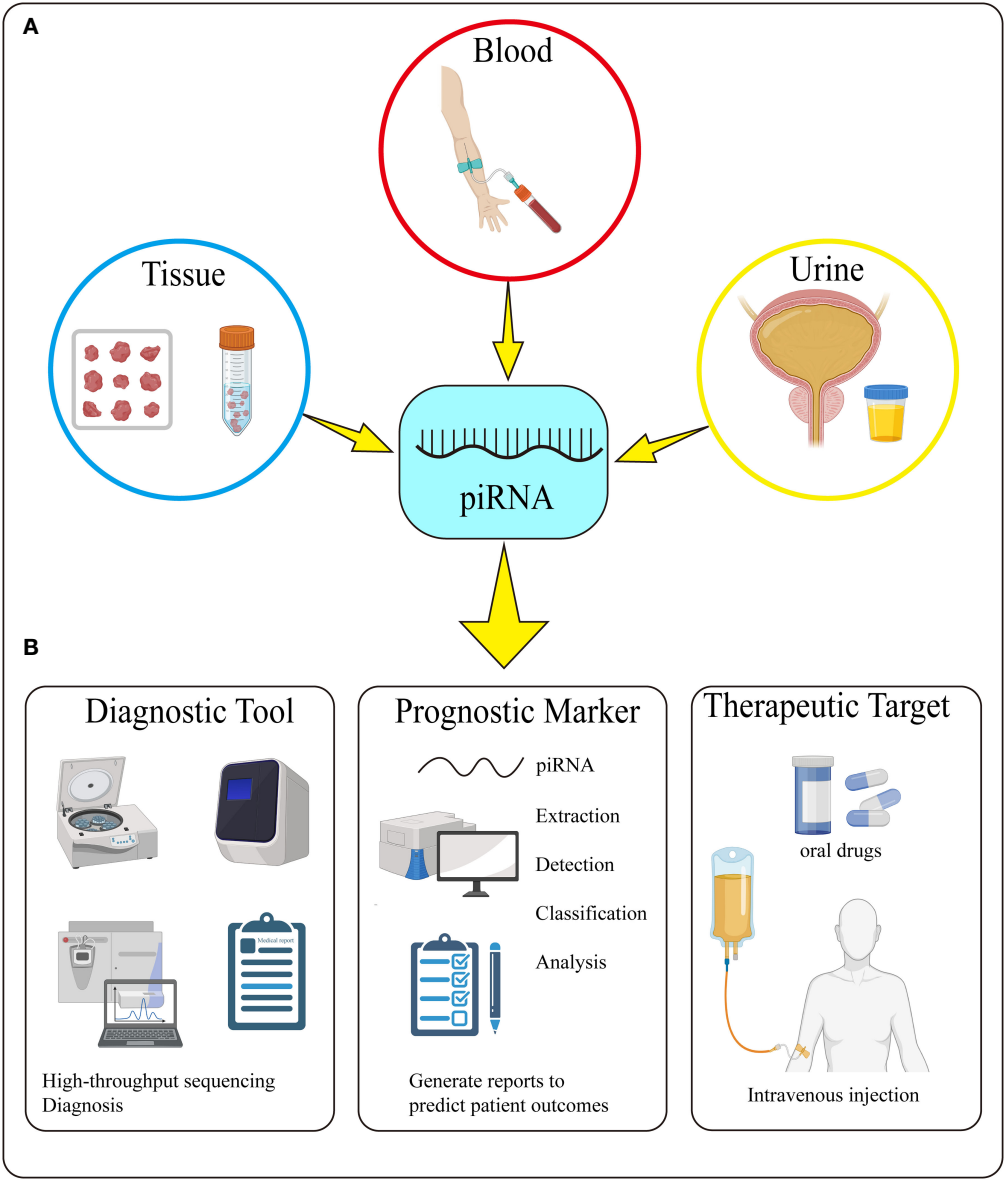
Detection and Potential Application of piRNA. **(A)** piRNA can be detected in tissue, blood, and urine. **(B)** piRNA can be used as a diagnostic tool, prognostic marker and therapeutic target through its extraction, detection, classification and analysis.

The discovery of piRNA has opened up a new field for the research of non-coding small molecule RNA. So far, number of scholars have predicted the application of piRNAs in biomedicine and have shown some promising results, it has a great prospect as a diagnostic tool and prognostic marker for urologic tumors in the future. Now we can diagnose tumors through liquid biopsy techniques such as CTCs, ctDNA and EVs, but no one has combined these techniques with piRNAs. If they are detected together with piRNAs, they may have more accurate diagnosis and prognosis. In addition, piRNAs also have the potential to open the door to predict treatment response. Many prostate cancer patients treated with novel ARTAs, PARP inhibitors or immune checkpiont inhibitors (112–114). The above liquid biopsy can also be used to predict treatment response, but it is still not accurate enough. We can also detect liquid biopsy molecules and piRNA together which may be more sensitive to predict treatment response. FGFR3 mutations selectively identified patients with favorable bladder cancer at radical cystectomy, whereas p53 and Ki-67 were only associated with unfavorable tumor features (115). We can combine the differential expression of piRNAs in bladder cancer to make a more accurate judgment of the prognosis of patients (**Figure 4**).

There are still many problems to be further studied. The synthesis of piRNAs is extremely complex. Many genomic regions filled with H3K9me3 markers neither bind Rhino nor transcribe piRNAs. What are their functions? In terms of piRNAs research, piRNAs expression in somatic cells and body fluids is easily distorted by background noise and technical artifacts. At present, there are also many deficiencies in the connection between piRNAs and urological tumors. For example, some piRNAs (such as piR-832) are highly expressed in tumor tissues of cancer patients, but their expression in body fluids is opposite. What is the reason for this differential expression? Moreover, many researchers only study the abnormal expression of piRNAs in certain tumor tissue but ignore the comparison with the abnormal expression in other tumor tissues, so there is no specificity when this piRNAs are used as a biomarker of tumors.

In the future, with the efforts of scientists and the application of new methods, more piRNAs will be identified, and the mechanism and clinical application of piRNAs in tumor progression will certainly be at the forefront of cancer research.

## Author contributions

JZ, WZ drafted the manuscript and prepared the figures and tables. YL prepared the figures and tables and polished the article. MP, YJ, AA, SM, HC collected the references and revised the manuscript. SS provided great help in major revisions. YR, WL, XY, ZC and YY participated in the discussion and gave constructive comments. All authors contributed to the article and approved the submitted version.
